# Comment on “Reproductive Outcomes of Transferring Blastocysts Derived From Frozen–Thawed Cleavage Embryos: A Systematic Review and Meta‐Analysis”

**DOI:** 10.1002/rmb2.12696

**Published:** 2025-11-17

**Authors:** Bangbei Wan, Weiying Lu

**Affiliations:** ^1^ Reproductive Medical Center, Hainan Women and Children's Medical Center Haikou China; ^2^ Department of Urology Haikou Affiliated Hospital of Central South University Xiangya School of Medicine Haikou China

**Keywords:** assisted reproductive techniques, blastocyst transfer, cryopreservation, embryo culture techniques, embryo transfer

## Abstract

Tran et al. synthesized seven studies (*n* = 2,057) comparing frozen–thawed cleavage embryos cultured to blastocyst then transferred (FT‐CDB) with direct frozen–thawed blastocyst transfer (DFB) in vitrification cycles. They found higher odds of clinical pregnancy and live birth with FT‐CDB and no difference in neonatal birth weight. We discuss implications for laboratories weighing thaw‐and‐extend versus direct blastocyst transfer, emphasizing estimands per oocyte retrieval/intention‐to‐treat, workflow metrics, and laboratory‐level covariates. Context from randomized controlled trials (RCTs) and systematic reviews suggests cumulative live birth may converge, highlighting the need for target‐trial emulation and transparent reporting to inform clinical decision‐making and guideline development.

Tran et al. synthesized seven studies (*n* = 2,057) comparing frozen–thawed cleavage embryos cultured to blastocyst then transferred (FT‐CDB) versus direct frozen–thawed blastocyst transfer (DFB) in vitrification cycles, and reported higher odds of clinical pregnancy and live birth with FT‐CDB, with no difference in neonatal birth weight; publication bias risk was low and study quality high [1]. These are decision‐relevant data for laboratories weighing thaw‐and‐extend workflows against direct blastocyst transfer.

## 1 How the New Meta‐Analysis Fits With the Totality of Evidence

Randomized controlled trials (RCTs) and systematic reviews consistently show that blastocyst‐stage transfers improve outcomes per fresh transfer, but advantages attenuate when outcomes are measured per retrieval/intention‐to‐treat because of higher cancellation and fewer supernumerary embryos with extended culture (Cochrane 2016; updated 2022). Recent RCTs and meta‐analyses focusing on single‐embryo transfer (SET) confirm higher ongoing pregnancy/delivery with single blastocysts versus single cleavage‐stage embryos in fresh cycles [2], whereas a 2024 multicentre RCT found no difference in cumulative live birth per retrieval (58.9% vs. 58.4%) despite a higher fresh‐transfer live birth and fewer transfers in the blastocyst arm; importantly, moderate preterm birth was higher after blastocyst transfer, underscoring the need to balance efficiency and perinatal safety [3]. Data from a 2024 multicentre RCT involving patients undergoing first‐time intracytoplasmic sperm injection (ICSI) showed trends favoring blastocysts per transfer but no statistically significant differences in implantation, clinical pregnancy, or live birth—again highlighting imprecision and context dependence [4].

In frozen cycles, embryo stage intersects with pregnancy loss phenotypes: a large frozen embryo transfer (FET) cohort suggested higher biochemical and clinical loss with Day‐3 transfers versus Day‐5—modulated by blastocyst morphology and Day (5 vs. 6)—implying that part of the stage effect is quality‐mediated rather than stage per se [5].

## 2 Points to Clarify to Aid Translation

First, estimands and denominators. Early embryology outcomes (implantation, biochemical pregnancy) are often reported per transfer, but policy decisions hinge on per retrieval/intention‐to‐treat (counting cancellations when thawed cleavages fail to reach blastocyst). We encourage Tran et al. to add absolute risk differences per retrieval alongside per‐transfer odds ratios and to tabulate the ratio of thawed Day 3 embryos to usable blastocysts and the share re‐frozen—metrics repeatedly identified as drivers of embryo utilization and throughput in Cochrane updates [6, 7].

Second, sources of heterogeneity. Prespecifying lab‐level covariates—thaw temperature profiles, artificial shrinkage, time‐lapse triage, and double‐freeze exposure—would help explain residual heterogeneity and align with best‐practice reporting emphasized in contemporary trials [3, 4].

Third, subgroup consistency. Given that single‐embryo and fresh‐transfer contexts often drive the apparent advantage of blastocysts [2], while cumulative outcomes can converge [3], subgroup analyses by indication (e.g., recurrent implantation failure), embryo quality/timing of blastocyst development (Day 5 vs. Day 6), and transfer policy (SET vs. ≤ double‐embryo transfer) would pinpoint where the benefit of FT‐CDB is greatest and where DFB remains equivalent.

## 3 Practice Implications and Next Steps

Taken together, the synthesis by Tran et al. supports FT‐CDB as a rational default when laboratories can absorb the in‐lab attrition and seek per‐transfer gains—while acknowledging that per‐retrieval cumulative live birth may be unchanged and that perinatal signals (e.g., moderate preterm) warrant monitoring [3]. We suggest that future RMB reports (i) co‐report per‐transfer and per‐retrieval outcomes with cancellations; (ii) disclose thaw‐and‐extend workflow metrics (how many thawed; how many reached blastocyst; how many transferred/re‐frozen); and (iii) emulate a target trial where randomization is infeasible, to minimize indication and immortal‐time biases—an approach emphasized across Cochrane updates [7].

## Ethics Statement

The authors have nothing to report.

## Consent

The authors have nothing to report.

## Conflicts of Interest

The authors declare no conflicts of interest.

## Data Availability

Data sharing not applicable to this article as no datasets were generated or analysed during the current study.
